# Secondary Syphilis with Eosinophilia Complicated by Severe Jarisch–Herxheimer Reaction

**DOI:** 10.1155/2020/2150314

**Published:** 2020-01-16

**Authors:** Anthony Elias, Kirsty Wark, Dana Slape, David Cook, Kenneth Lee, Genevieve Mckew

**Affiliations:** ^1^Department of Microbiology & Infectious Diseases, Concord Repatriation General Hospital, Sydney, New South Wales, Australia; ^2^Department of Dermatology, Concord Repatriation General Hospital, Sydney, New South Wales, Australia; ^3^Department of Anatomical Pathology, Concord Repatriation General Hospital, Sydney, New South Wales, Australia

## Abstract

It has long been acknowledged that syphilis is a disease with a diverse range of presentations. We herein describe a case of a young man who presented with fever, rash, and eosinophilia following the commencement of allopurinol, only to be diagnosed with secondary syphilis on histopathology. His treatment was complicated by a severe exacerbation of his cutaneous eruption following the commencement of penicillin, likely secondary to a Jarisch–Herxheimer reaction, an entity often overlooked by clinicians managing syphilis.

## 1. Introduction

In recent years, syphilis has experienced a resurgence following a dramatic reduction in incidence throughout the twentieth century. Lack of exposure to its various manifestations has resulted in difficulties in diagnosis of once a well-recognised disease. Here, we report a case of secondary syphilis complicated by the development of a likely Jarisch–Herxheimer reaction following the administration of penicillin. The diagnosis was clouded somewhat by the possibility of a drug reaction with eosinophilia and systemic symptoms (DRESS) syndrome following commencement of allopurinol. It serves as a reminder of this phenomenon, which though has long been well described, remains poorly understood.

### 1.1. Case Report

A 26-year-old MSM of Indigenous Australian and Pacific Islander descent presented with a two week history of fevers, malaise, and rash. This was on a background of previous treatment with three IM benzathine penicillin injections weekly for latent syphilis 2 years earlier. One month prior to his presentation, he had developed inflammation of the first metatarsophalangeal joint on his right foot. He was diagnosed with gout and treated with ibuprofen before being commenced on allopurinol. Approximately one week later, he developed lethargy, a dry cough, pharyngitis, and otalgia, for which he was prescribed a five-day course of roxithromycin by his general practitioner for a presumed upper respiratory tract infection. His symptoms however progressed further over the next two weeks, developing fevers, night sweats, headaches, myalgias, nausea, vomiting, and a nonpruritic macular rash starting centrally on the trunk and face before spreading peripherally. He reported a new male sexual contact in the past few months and sought STI testing. His RPR was 1 : 8, and HIV serology was negative. He self-ceased allopurinol days prior his presentation as his symptoms failed to improve.

On admission he was febrile up to 41°C and tachycardic. Examination revealed bilateral cervical and supraclavicular lymphadenopathy, mild pharyngitis, and macular rash over his face, scalp, upper arms, and torso. His initial investigations showed a normal white cell count with elevated eosinophils of 0.7 × 10^9^/L, CRP 20 mg/L, as well as raised liver enzymes with an ALP 195 U/L, GGT 234 U/L, and AST 150 U/L. An abdominal ultrasound showed periportal and supraclavicular lymphadenopathy and borderline splenomegaly (15 cm). Repeat RPR was 1 : 8.

He was administered benzathine penicillin 1.8 g IM with concurrent prednisone 60 mg as treatment for secondary syphilis before commencing regular prednisone 50 mg daily on the possibility the presentation could be related to DRESS from allopurinol.

**Figure 1:**
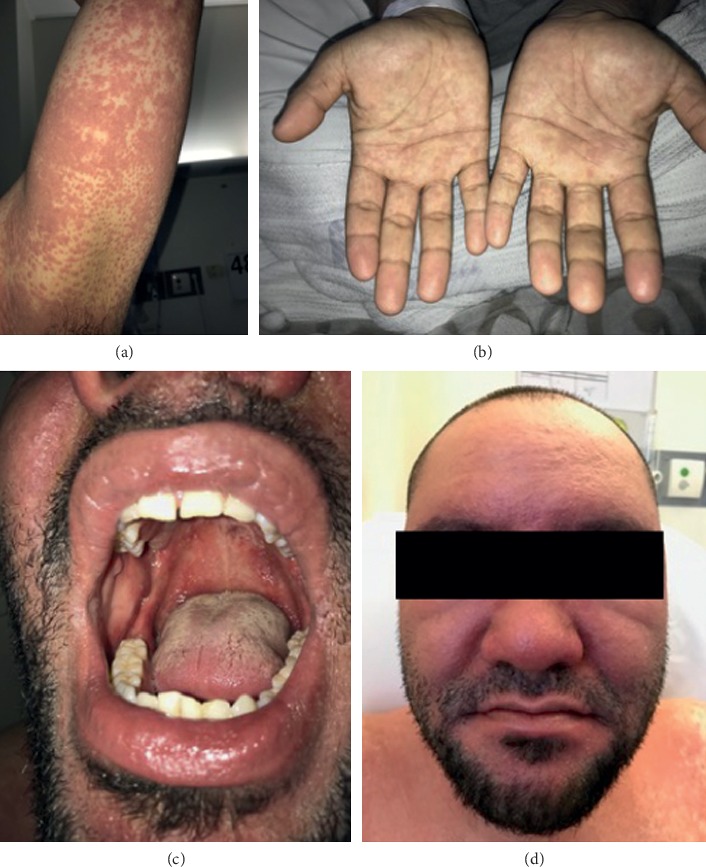
(a) Erythematous confluent macular and papular follicular eruption on his left upper arm. (b) The eruption involving the acral surfaces. (c) Marked facial swelling. (d) Oral mucosa ulcers.

The following day his rash had progressed, becoming more confluent and spreading to involve his distal upper and lower limbs with profound perifollicular accentuation (Figure 1(a)). His palms also revealed faint macular erythema (Figure 1(b)). He also developed marked facial swelling (Figure 1(c)) as well as petechial changes over his hard palate with associated ulceration of his oral mucosa (Figure 1(d)).

**Figure 2:**
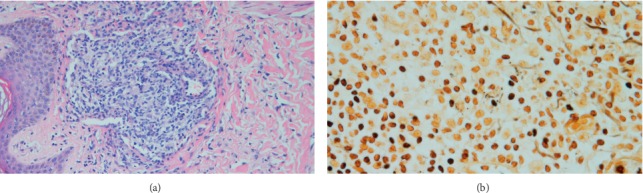
(a) Lymphohistiocytic infiltrate surrounding superficial dermal vessels and histiocytes aggregating into poorly formed granulomas (×100 magnification). (b) Warthin–Starry special stain showing numerous spirochaetes amongst the lymphohistiocytic infiltrate (×400 magnification).

A skin biopsy showed a dermal lymphoplasmacytic and histiocytic infiltrate, with the inflammation predominantly surrounding the vessels and hair follicles. There were aggregates of histiocytes leading to the formation of poorly formed granulomas (Figure 2(a)). Scant eosinophils were seen. A Warthin–Starry stain was performed showing moderate numbers of spiral and curved bacilli within histiocytes, features in keeping with spirochaetes (Figure 2(b)).

Over the next few days, the patient significantly improved with no further fevers within 24 hours of penicillin administration, and facial erythema and oedema settled after 72 hrs. The rash gradually settled; however, his eosinophils continued to rise over the next week, peaking at 2.5 × 10^9^/L. He was discharged with a weaning prednisone regime.

On follow-up in a local clinic four weeks later, his rash had resolved, his eosinophilia normalised, and a repeat RPR (at a different laboratory) was 1 : 32. HLA-B 58 : 01 was not detected.

## 2. Discussion

Syphilis is an infection caused by the bacteria *Treponema pallidum*, usually transmitted through contact with an infectious lesion during sexual activity. The most common manifestation is primary syphilis, characterised by the development of a chancre at the inoculum site. Of those who develop this manifestation, if left untreated, approximately 25% go on to develop secondary syphilis [1], a diffuse inflammatory reaction to haematogenous spread of spirochaetes. This is characterised by the development of a cascade of systemic symptoms including fevers, malaise, headache, anorexia, pharyngitis, myalgias, diffuse lymphadenopathy, and skin manifestations.

Secondary syphilis has several cutaneous manifestations, which contribute to its reputation of “the great imitator.” It is usually a generalised symmetrical erythematous eruption, with macular and papular morphology, often involving acral surfaces. Condyloma lata may be seen on genital, anal, or oral mucosal surfaces. A chancre may still be present [2]. Syphilis may also present with a pustular, pustulonodular, or framboesiform eruption [3]. Hair may be also involved with a patchy or moth-eaten pattern of alopecia [4].

Histopathologically, it is most commonly associated with a lichenoid and/or superficial and deep perivascular inflammatory pattern. The epidermis may be acanthotic and parakeratotic. There are often endothelial swelling, perineural invasion of the inflammatory infiltrate, plasma cell infiltrate, and intraepidermal neutrophils [5].

Numerous other clinical features that may be seen in secondary syphilis include synovitis/periosteitis and rarely meningitis. Eye disease can also be observed with the most common manifestations being anterior and/or posterior uveitis.

In terms of laboratory investigations, hepatitis is regularly seen, with the most frequently observed abnormality being a raised ALP. There is limited data reporting peripheral eosinophilia in cases of secondary syphilis, though there have been reports of eosinophil-rich skin lesions [6]. A transient subnephrotic proteinuria is often observed; however, rarely the renal injury seen is more severe with membranous glomerulonephritis being the most common glomerulopathy [7].

It is generally self-limiting, settling over 3–6 weeks, though those left untreated go on to develop latent syphilis with a significant percentage going on to develop tertiary syphilis.

In approximately 10–35% of those treated for syphilis [8, 9], treatment is complicated by the development of the Jarisch–Herxheimer reaction, an inflammatory reaction typically seen in the first 24 hrs following commencement of treatment.

The underlying mechanism remains poorly understood but is believed to relate to cytokine and lipopolysaccharide release from lysed spirochaetes resulting in recruitment of phagocytes [10]. It is characterised by the development of fevers and systemic symptoms including headaches, fevers, myalgias, and an exacerbation of the symptoms and signs of the disease, in particular skin rashes. The reaction is usually transient, settling after 24 hrs.

Histologically, the reaction displays acute vascular congestion in the capillaries and small blood vessels resulting in surrounding connective tissue oedema and neutrophilic infiltrates [11]. Rarely, the reaction can be more serious, being associated with increased uterine contractions, preterm labour, preterm delivery, or fetal death in pregnancy [12] and with case reports of serious reactions observed in those treated for neurosyphilis [13], ocular syphilis [14], and aortitis [15].

The reaction seemed to be more frequently observed in younger patients, cases of early syphilis, and those with higher RPRs (>1 : 32) [9]. Of those with late syphilis, though uncommon, the reaction is more frequently observed in the first dose of penicillin, compared with subsequent doses, adding weight to the lysed-spirochaete hypothesis. It appears both the type of drug and the rate of spirochaete clearance from the body have little influence on the incidence of the reaction [10].

Management of the Jarisch–Herxheimer reaction is generally supportive, with limited evidence for pharmacologic intervention. However, there has been some data suggesting that pretreatment with steroids, in the form of prednisolone 40–60 mg/day for three days commenced 24 hours prior to antibiotic administration, may prevent the development of fever in patients treated for early syphilis [16]. There has been growing evidence that anti-TNF-alpha antibody therapy has been shown to reduce both frequency and severity of the Jarisch–Herxheimer reaction in relapsing fever [17].

The case above describes a presentation of secondary syphilis diagnosed on histopathology, complicated by a likely Jarisch–Herxheimer reaction following the administration of penicillin. What was unusual in this presentation was the marked eosinophilia, which in association with the clinical syndrome and the recent commencement of allopurinol raises the possibility of DRESS syndrome.

DRESS syndrome is a rare, severe, delayed drug-induced reaction which is characterised by cutaneous, haematological, and multiorgan involvement [18]. It has been more commonly associated with the use of antiepileptic drugs, allopurinol, sulphonamides, and olanzapine, though has been reported with many other medications [19]. HLA B∗58:01 phenotype is associated with an increased risk of DRESS to allopurinol in patients of Han-Chinese background.

Histopathologically, it is commonly associated with interface dermatitis, parakeratosis, and a predominantly superficial and perivascular dermal polymorphous inflammatory infiltrate. Eosinophils and atypical lymphocytes are occasionally seen. However, multiple inflammatory patterns can be present in the same specimen, which is more characteristic of DRESS than in comparison to non-DRESS macular-papular eruptions [20].

Whilst the patient fulfilled the RegiSCAR criteria for probable DRESS [21], the presence of a histopathologically confirmed alternative diagnosis makes this exceedingly unlikely. This highlights the importance of excluding other clinical entities before diagnosing DRESS on criteria alone. It is worth noting, however, that infections have been implicated as triggers of DRESS, with an association observed between reactivation of Epstein–Barr virus and human herpes virus 6 and the development of DRESS [18].

## 3. Conclusion

Though there remains a degree of uncertainty as to whether the clinical manifestations observed could all be attributed to secondary syphilis, the marked exacerbation of the cutaneous eruption following penicillin demonstrates the importance of considering the Jarisch–Herxheimer reaction following the commencement of treatment for syphilis. Despite being a common phenomenon, the Jarisch–Herxheimer reaction remains often overlooked, as the definition is nonspecific and the symptoms are often present to some degree prior to antibiotic administration. It is essential clinicians are aware this may occur and can advise patients prior to treatment.

## References

[1] Clark E. G., Danbolt N. (1964). The Oslo study of the natural course of untreated syphilis: an epidemiologic investigation based on a re-study of the Boeck-Bruusgaard material. *Medical Clinics of North America*.

[2] Baughn R. E., Musher D. M. (2005). Secondary syphilitic lesions. *Clinical Microbiology Reviews*.

[3] Balagula Y., Mattei P. L., Wisco O. J., Erdag G., Chien A. L. (2014). The great imitator revisited: the spectrum of atypical cutaneous manifestations of secondary syphilis. *International Journal of Dermatology*.

[4] Dylewski J., Duong M. (2007). The rash of secondary syphilis. *Canadian Medical Association Journal*.

[5] Hoang M. P., High W. A., Molberg K. H. (2004). Secondary syphilis: a histologic and immunohistochemical evaluation. *Journal of Cutaneous Pathology*.

[6] Requena L., Kutzner H., Palmedo G. (2005). Histiocytoid sweet syndrome: a dermal infiltration of immature neutrophilic granulocytes. *Archives of Dermatology*.

[7] Hunte W., Al-Ghraoui F., Cohen R. J. (1993). Secondary syphilis and the nephrotic syndrome. *Journal of the American Society of Nephrology: JASN*.

[8] Miller W. M., Gorini F., Botelho G. (2010). Jarisch-Herxheimer reaction among syphilis patients in Rio de Janeiro, Brazil. *International Journal of STD & AIDS*.

[9] Yang C. J., Lee N. Y., Lin Y. H. (2010). Jarisch-herxheimer reaction after penicillin therapy among patients with syphilis in the era of the HIV infection epidemic: incidence and risk factors. *Clinical Infectious Diseases*.

[10] Pound M. W., May D. B. (2005). Proposed mechanisms and preventative options of Jarisch-Herxheimer reactions. *Journal of Clinical Pharmacy and Therapeutics*.

[11] Sheldon W. H., Heyman A. (1949). Morphologic changes in syphilitic lesions during the Jarisch-Herxheimer reaction. *American Journal of Syphilis, Gonorrhea, and Venereal Diseases*.

[12] Klein V. R., Cox S. M., Mitchell M. D., Wendel J. G. (1990). The Jarisch-Herxheimer reaction complicating syphilotherapy in pregnancy. *Obstetrics and Gynecology*.

[13] Butler T. (2017). The Jarisch-Herxheimer reaction after antibiotic treatment of spirochetal infections: a review of recent cases and our understanding of pathogenesis. *The American Journal of Tropical Medicine and Hygiene*.

[14] Fathilah J., Choo M. M. (2003). The Jarisch-Herxheimer reaction in ocular syphilis. *The Medical Journal of Malaysia*.

[15] Hughes G. R. (1968). Jarisch-Herxheimer reaction and syphilitic aortitis. *BMJ*.

[16] Gudjónsson H., Skog E. (1968). The effect of prednisolone on the Jarisch-Herxheimer reaction. *Acta Dermato-Venereologica*.

[17] Fekade D., Knox K., Hussein K. (1996). Prevention of Jarisch-Herxheimer reactions by treatment with antibodies against tumor necrosis factor *α*. *New England Journal of Medicine*.

[18] Walsh S. A., Creamer D. (2011). Drug reaction with eosinophilia and systemic symptoms (DRESS): a clinical update and review of current thinking. *Clinical and Experimental Dermatology*.

[19] Kardaun S. H., Sekula P., Valeyrie-Allanore L. (2013). Drug reaction with eosinophilia and systemic symptoms (DRESS): an original multisystem adverse drug reaction: results from the prospective RegiSCAR study. *British Journal of Dermatology*.

[20] Ortonne N., Valeyrie-Allanore L., Bastuji-Garin S. (2015). Histopathology of drug rash with eosinophilia and systemic symptoms syndrome: a morphological and phenotypical study. *British Journal of Dermatology*.

[21] Peyriere H., Dereure O., Breton H. (2006). Variability in the clinical pattern of cutaneous side-effects of drugs with systemic symptoms: does a DRESS syndrome really exist?. *British Journal of Dermatology*.

